# Fabrication of calcium phosphate 3D scaffolds for bone repair using magnetic levitational assembly

**DOI:** 10.1038/s41598-020-61066-3

**Published:** 2020-03-04

**Authors:** Vladislav A. Parfenov, Vladimir A. Mironov, Elizaveta V. Koudan, Elizaveta K. Nezhurina, Pavel A. Karalkin, Frederico DAS Pereira, Stanislav V. Petrov, Alisa A. Krokhmal, Timur Aydemir, Igor V. Vakhrushev, Yury V. Zobkov, Igor V. Smirnov, Alexander Yu. Fedotov, Utkan Demirci, Yusef D. Khesuani, Vladimir S. Komlev

**Affiliations:** 1 Laboratory for Biotechnological Research “3D Bioprinting Solutions”, Moscow, Russia; 2 A.A. Baikov Institute of Metallurgy and Material Science, Russian Academy of Sciences, Moscow, Russia; 3 P.A. Hertsen Moscow Oncology Research Center - branch of National Medical Research Radiological Center, Moscow, Russia; 4 V.N. Orekhovich Institute of Biomedical Chemistry, Moscow, Russia; 5 Stanford University, Department of Radiology, Stanford, CA USA

**Keywords:** Biomedical materials, Tissue engineering

## Abstract

The calcium phosphate particles can be used as building blocks for fabrication of 3D scaffolds intended for bone tissue engineering. This work presents for the first time a rapid creation of 3D scaffolds using magnetic levitation of calcium phosphate particles. Namely, tricalcium phosphate particles of equal size and certain porosity are used, which undergo the process of recrystallization after magnetic levitational assembly of the scaffold to ensure stitching of the scaffold. Label-free levitational assembly is achieved by using a custom-designed magnetic system in the presence of gadolinium salts, which allows the levitation of calcium phosphate particles. Chemical transformation of tricalcium- to octacalcium phosphate under the condition of magnetic levitation in non-homogeneous magnetic field is also demonstrated. This approach allows obtaining rapidly the octacalcium phosphate phase in the final 3D product, which is biocompatible.

## Introduction

Bone is a mineral-organic composite and mainly consists of calcium phosphate (CP) and also includes proteins (mainly collagen type I), and water. In bone tissue, CP is presented as nonstoichiometric and partially crystallized apatite^1^. Thus, the application of 3D scaffold based on CP is highly promising for bone tissue engineering as a scaffold material. There are a number of advantages for using CP as bone graft materials such as biodegradation, biocompatibility and osteoconductive properties. Most of synthetic CP materials used in clinical practice based on tricalcium phosphate (TCP) ceramic, which is a reliable and osteoconductive. TCP is commercially available in form of granules, cements, or blocks^2–4^. However, the rate of biodegradation is often lower than the rate of osteogenesis, which leads to walling up of CP materials in the area of the bone defect^5^. In this regard, the biomimetic approach of using octacalcium phosphate (OCP) as a possible precursor to HA in natural bone tissue has been proposed^6,7^. It is worth mentioning that production of sufficient quantities of OCP is very challenging and usually takes a couple of weeks^8^. Accordingly, the development of technology for robust preparation of OCP phase is now an important task for manufacturing of scaffolds intended for bone grafting.

In the past decade, a several number of techniques for rapid prototyping of 3D bone grafts based on ceramics have been developed and widely applied^9–12^. Rapid prototyping is an additive approach based on layer by layer fabrication of 3D objects and includes several steps: (i) the creation of computational model of the desired object; (ii) 3D slicing of the model; (iii) the layer-by-layer fabrication of the object with the required size, shape and inner structure *via* different 3D printing approaches such as laser stereolithography^13^, selective laser sintering^14^, extrusion-based printing^8^, inkjet-based printing^15,16^. There are few most useful techniques for CP ceramics additive production. The most frequently used techniques is mixing of ceramic particles with polymers followed by their 3D binding, and further high-temperature treatment^11,17^. Another route based on inverse matrix-negative 3D printing, its filling with CP slurry, and heat processing for burning out the negative^10^. Selective laser sintering is a widespread 3D route for production of CP scaffolds, which is based on the powder coating by polymer that evaporates after the laser explosion^14^. Despite this, additive approach has some limitations such as limit of tool resolution, necessity for supportive material, shrinkage of ceramics during high temperature processing and etc.

As an alternative to additive approach, the formative technology, which engages the simultaneous assembly in the whole volume of fabrication chamber has arisen recently. In the formative approach the shape and the microstructure of the scaffold are determined by physical forces such as acoustic waves, magnetic and electric fields. The desired geometry of 3D scaffold in accordance with preliminary 3D model is achieved by calculated and modelled certain distribution of physical fields in 3D space. Thus, magnetic field is the most commonly used for levitation and modification of different objects (magnetic levitation (MagLev)). To provide the condition for levitation of diamagnetic objects for magnetic assembly, the paramagnetic medium, e.g. with gadolinium salts, could be added. Gadolinium-based (Gd^3+^-based) pharmaceuticals, commonly used *via* MagLev approach, are FDA approved for clinical use as MRI contrast agents and consequently comprise non-toxic agents The magnetic levitational assembly has been proposed recently as a novel way to fabricate biomaterials, scaffolds for tissue engineering^18–21^. The magnetic fabrication of viable chondrospheres-based scaffolds *via* levitation has been demonstrated in our previous study^22^.

Theoretically, magnetic levitational approach can be used to assemble spatial 3D scaffolds from single diamagnetic CP granules. However, to compensate for gravity, levitation of CP requires several times greater magnetic force than levitation of living cells due to material density difference. This problem can be solved by using higher magnetic field gradient, which can be achieved by application of either superconductive electromagnets (or Bitter electromagnets) or higher concentrations of paramagnetic salts in medium. The main concept of our work is based on providing assembly of 3D scaffolds from TCP particles under levitation conditions with formation of OCP phase in the final product, excluding the use of any temporal basement, nozzles and magnetic labels. We hypothesize that it will be possible to fabricate more advanced and biodegradable scaffolds with improved properties under the condition of magnetic levitation. In this study, for the first time, we present successful fabrication *via* formative technology of biocompatible scaffold for the purpose of bone tissue engineering.

## Results

### The production of α-TCP particles

Scanning electron micrographs of initial α-TCP ceramic particles intended for fabrication of a CP-based 3D scaffold in a magnetic field are shown in Fig. S1 (Supporting Information). The average size of α-TCP particles was about 250–500 μm. The resulting α-TCP crystals had a globular-like morphology with maximum diameter of up to several micron.

### Computer simulation of magnetic field and assembling of particles

**Figure 1:**
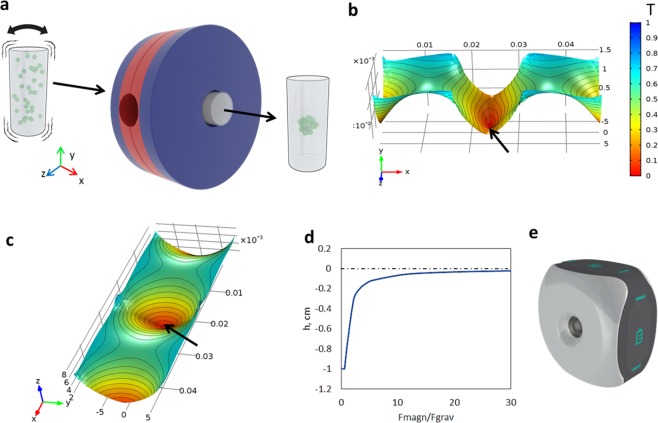
The magnetic setup. (**а**) Schematic showing of installation and scaffold fabrication. (**b**) The magnetic field on the horizontal axial section (the arrows show the magnetic well). (**c**) A magnetic field on the vertical axial section. (**d**) Dependence of distance from levitating assembly to center of magnetic field on relation between magnetophoretic and gravity force acting on particles. (**e**) A model of magnetic printer.

**Figure 2:**
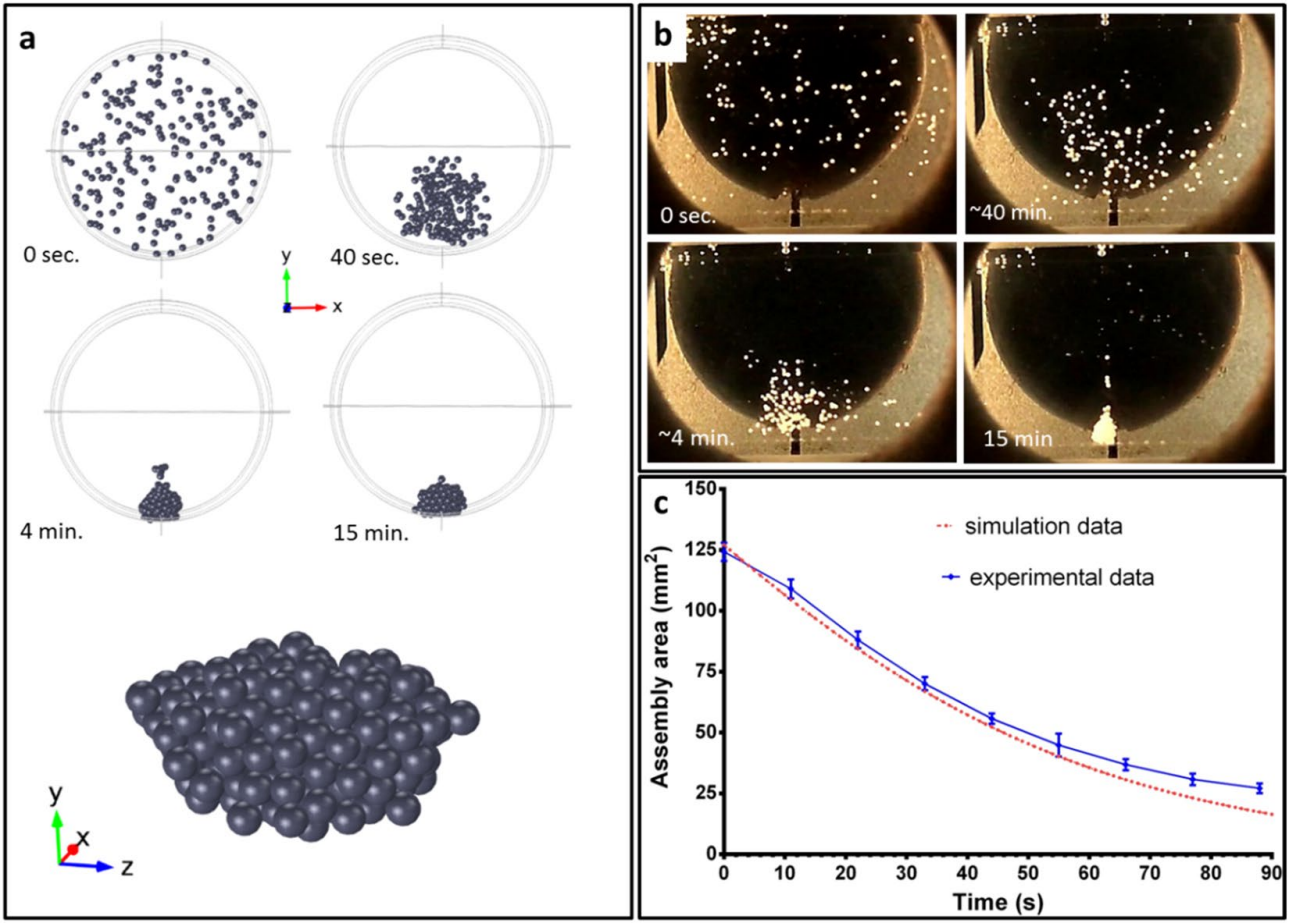
The levitational assembly of 3D scaffold based on CP in paramagnetic medium by using the magnetic field (the concentration of gadolinium salt is 3 M). (**a**) Simulation of assembly of α-TCP particles and shape of scaffold. (**b**) The dynamics of assembly of α-TCP particles. (**c**) The time-assembly area curves of simulation and experiment in red and blue, respectively.

To perform the assembly, we used the magnetic setup (Fig. 1a) which provides non-homogeneous magnetic field in the working area. Magnetic field used as a temporary scaffold in this experiment was modelled by COMSOL software. The distribution of the magnetic induction values in the vertical and horizontal section of working area is shown in the 3D model graph (Fig. 1b,c). The magnetic setup provides levitation of particles on the different height depending on the magnetophoretic force/gravity force ratio which in turn is regulated by concentration of paramagnetic salt in the medium (Fig. 1d). The external view of the magnetic setup is shown in the Fig. 1e. According to computer simulation the optimal concentration of paramagnetic salt in the buffer solution and the required configuration of magnetic field required in order to provide levitational assembly were determined. The simulation also allows evaluation the shape of the scaffold assembly time and the height of levitation (Fig. 2). The height of scaffold assembly depends on paramagnetic salt concentration in medium, and if it is high enough, then generated magnetophoretic force can overcome gravity and make granules float up. Due to non-constant gradient of the magnetic field in vertical direction, interaction between magnetic and gravity forces, both acting on the scaffold, also depends on the height of the assembly. Peak magnitude of magnetophoretic force is observed in the center of area inside magnets, but due to gravity the scaffold can be placed in the area of lower magnetic field. So, the magnitude of magnetophoretic force must exceed gravity force several times to hold the scaffold at some height above the bottom. The increase in this ratio will increase the height of the scaffold assembly position. It should be mentioned that accurate computer simulation requires accounting for the characteristics of fluid and particles. While the density could be defined quite easily, CP permeability was not known a priori. The viscosity of the medium in our setting was estimated experimentally: the motion of a granule was captured to define its velocity, and viscosity was calculated from the Stokes’ law. In our work the velocity was very low and thus Reynolds number was less than 1. In this case fluid resistivity was described by Stokes’ law quite accurately. Using all the other known parameters of CP and medium, magnetic permeability of the particles could be calculated from the comparison of assembly time in a pilot experiment and computer simulation. It was equal to *µ*_*p*_ = 0.99999, which is very close to bone’s magnetic permeability values^23^.

### Fabrication of scaffolds using magnetic levitational assembly

Although computer modelling predicted a spherical shape of the final scaffold (Fig. 2a), after its assembly vertical shape elongation was visualized (Fig. 2b). This shape could be explained by variation in particle density due to some differences in porosity. It also can be noted that after 20 h of levitation in buffer №1 particles started to settle down on the scaffold due to filling of pores. To compare the simulated and experimental processes of assembly, the visible area of CP conglomerate was measured. Results are shown in Fig. 2c. Experimentally measured area was declining till the particles were compactly packed, while the final area of the scaffold depended on the number of particles. The computer simulation of assembly was conducted for small number of particles, so the size of the final scaffold was comparable with granule size. Despite the difference in the final scaffold size between experiment data and computer simulation, the intermediate time dependence for conglomerate area in both cases was equal. Thus, the speed of particles convergence is not affected by number of particles and steadily decreases on time.

**Figure 3:**
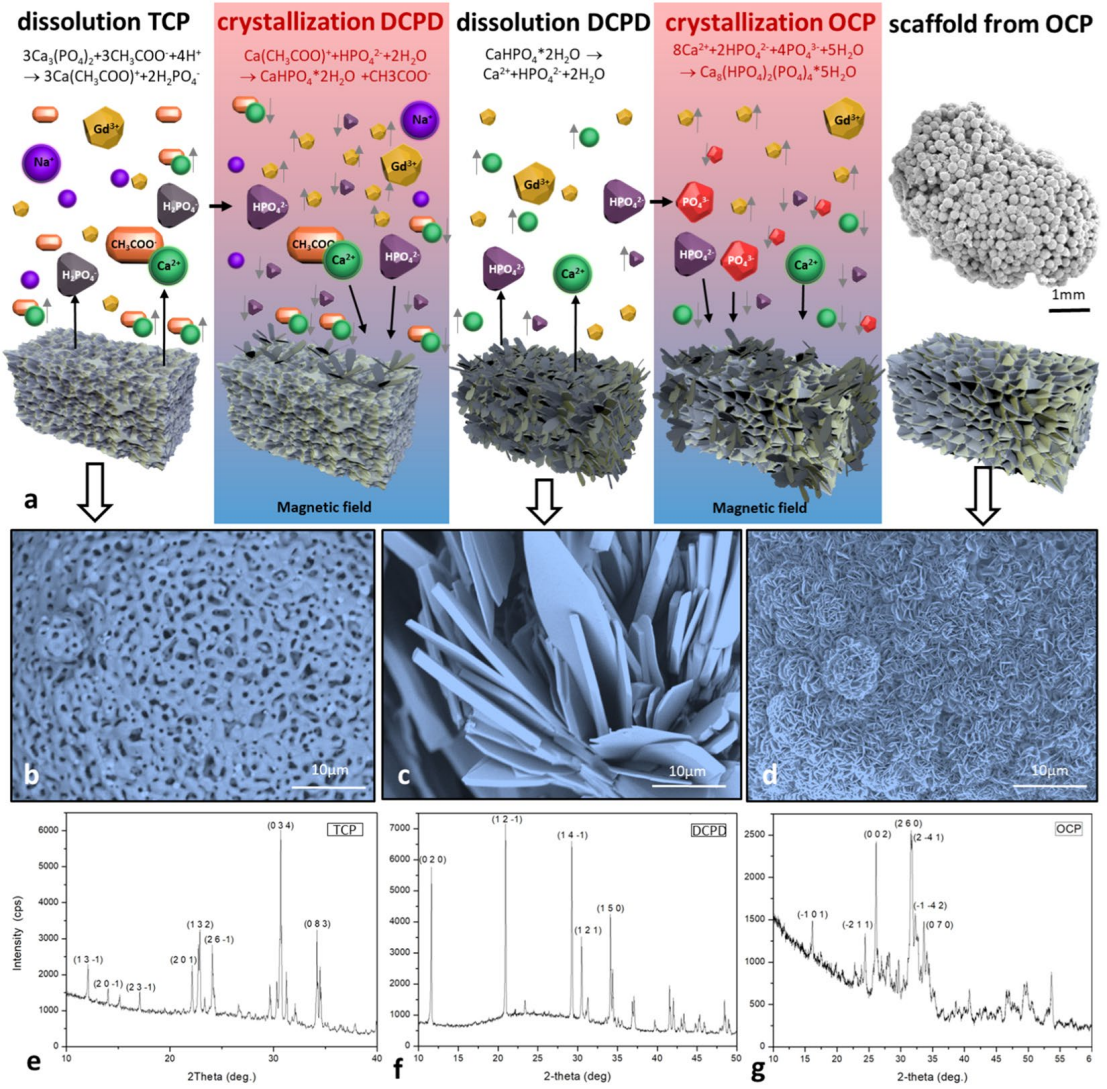
Fabrication of 3D scaffold based on CP in paramagnetic medium by using the magnetic field (the concentration of gadolinium salt is 3 M). (**а**) Schematic showing of recrystallization stages. (**b**) SEM image shows the surface of initial α-TCP particles (bar 10 μm). (**c**) SEM image shows the DCPD phase (bar 10 μm). (**d**) SEM image shows the surface of the obtained scaffold (OCP phase) (bar 10 μm). (**e**) X-ray phase analysis of α-TCP particles. (**f**) X-ray phase analysis of 3D scaffold after phase 1 of magnetic levitation fabrication (20 h in buffer № 1, RT). (**g**) X-ray phase analysis of 3D scaffold after phase 1 (20 h in buffer № 1, RT) and phase 2 (20 h in buffer № 2, RT) of magnetic levitation fabrication.

To facilitate recrystallization of the particles during the assembly, the scaffold levitated in the magnetic field for 40 h at RT. The process of transformation of α-TCP into OCP is shown in Fig. 3a. At the end of the phase 1 the initial α-TCP structure (Fig. 3b,e) recrystallized into dicalcium phosphate dihydrate (brushite, DCPD) (Fig. 3c,f). α-TCP on the surface of 3D scaffold started to dissolve in buffer №1 due to the interaction of the surface with the products of dissociation of sodium acetate accompanied by release of Ca^2+^ and H_2_PO_4_^−^ (predominates in the solution) and НPO_4_^2−^ in the solution. When the concentration of these ions in the interphase reached its certain critical value, the crystallization occurred. The level of saturation was enhanced by application of the non-homogeneous magnetic field since the concentration of anions and cations grew faster due to the effect of magnetic sedimentation. The increased concentration of ions in the solution led to the formation of DCPD crystals on the surface of α-TCP with their subsequent growth. The formation of DCPD occurred according to the following reaction:

1 $$\begin{array}{c}2{{\rm{Ca}}}_{3}{({{\rm{PO}}}_{4})}_{2}+2{{\rm{NaH}}}_{2}{{\rm{PO}}}_{4}+2{{\rm{CH}}}_{3}{\rm{COOH}}+12{{\rm{H}}}_{2}{\rm{O}}\\ \,=\,6{{\rm{CaHPO}}}_{4}\cdot 2{{\rm{H}}}_{2}{\rm{O}}+2{{\rm{CH}}}_{3}{\rm{COONa}}\end{array}$$

At the end of phase 2 the whole structure underwent the full transformation into OCP (Fig. 3d,g). Firstly, DCPD crystals on the surface of 3D scaffold obtained due to phase 1 began to dissolve in buffer № 2. The dissolution occurred due to the interaction of DCPD crystals with acetic acid and sodium hydroxide dissociation products which promote the increase of concentration of Ca^2+^ cations and H_2_PO_4_^−^ and НPO_4_^2−^ anions in the interphase. As well as in the phase 1, magnetic sedimentation promoted the increase of concentration of ions in the interphase which led to the deposition of OCP and formation of OCP crystals on the surface of DCPD. The OCP formation reaction can be expressed as follows:

2 $$\begin{array}{c}8{{\rm{CaHPO}}}_{4}\cdot 2{{\rm{H}}}_{2}{\rm{O}}+8{\rm{NaOH}}+4{{\rm{CH}}}_{3}{\rm{COOH}}\\ \,\to \,{{\rm{Ca}}}_{8}{({{\rm{HPO}}}_{4})}_{2}{({{\rm{PO}}}_{4})}_{4}\cdot 5{{\rm{H}}}_{2}{\rm{O}}+2{{\rm{Na}}}_{2}{{\rm{HPO}}}_{4}+4{{\rm{CH}}}_{3}{\rm{COONa}}+19{{\rm{H}}}_{2}{\rm{O}}\end{array}$$

Gibbs energies were calculated based on the standard activity of the ions presented in the solutions. *ΔG* of DCPD formation is −7 kJ mol^−1^, and *ΔG* of OCP formation is −663 kJ mol^−1^. It confirmed the possibility of these reactions.

SEM image of a 3D scaffold which was obtained under the similar buffer conditions but without application of the magnetic field (regarded as control sample) is shown in Fig. S2 (Supporting Information). On the surface of the control sample no OCP crystal formation was observed which can be attributed to the lack of magnetic field. Indeed, the levitational condition in the magnetic field facilitates the formation of OCP in a fairly short time, within 40 h.

SEM image of a 3D scaffold obtained in magnetic field is shown in Fig. S3 (Supporting Information). Porosity in the range about 57 to 65 vol. % is obtained. Dominant pore size is in the range 1–10 μm. These pores occupy about 53 vol. % of the total open pores within the scaffolds. Other pores are those of from 50 to 200 mm in diameter.

3D scaffold had slightly low compression strength (about 5 MPa) (Fig. S5).

### Biocompatibility of 3D scaffold

**Figure 4:**
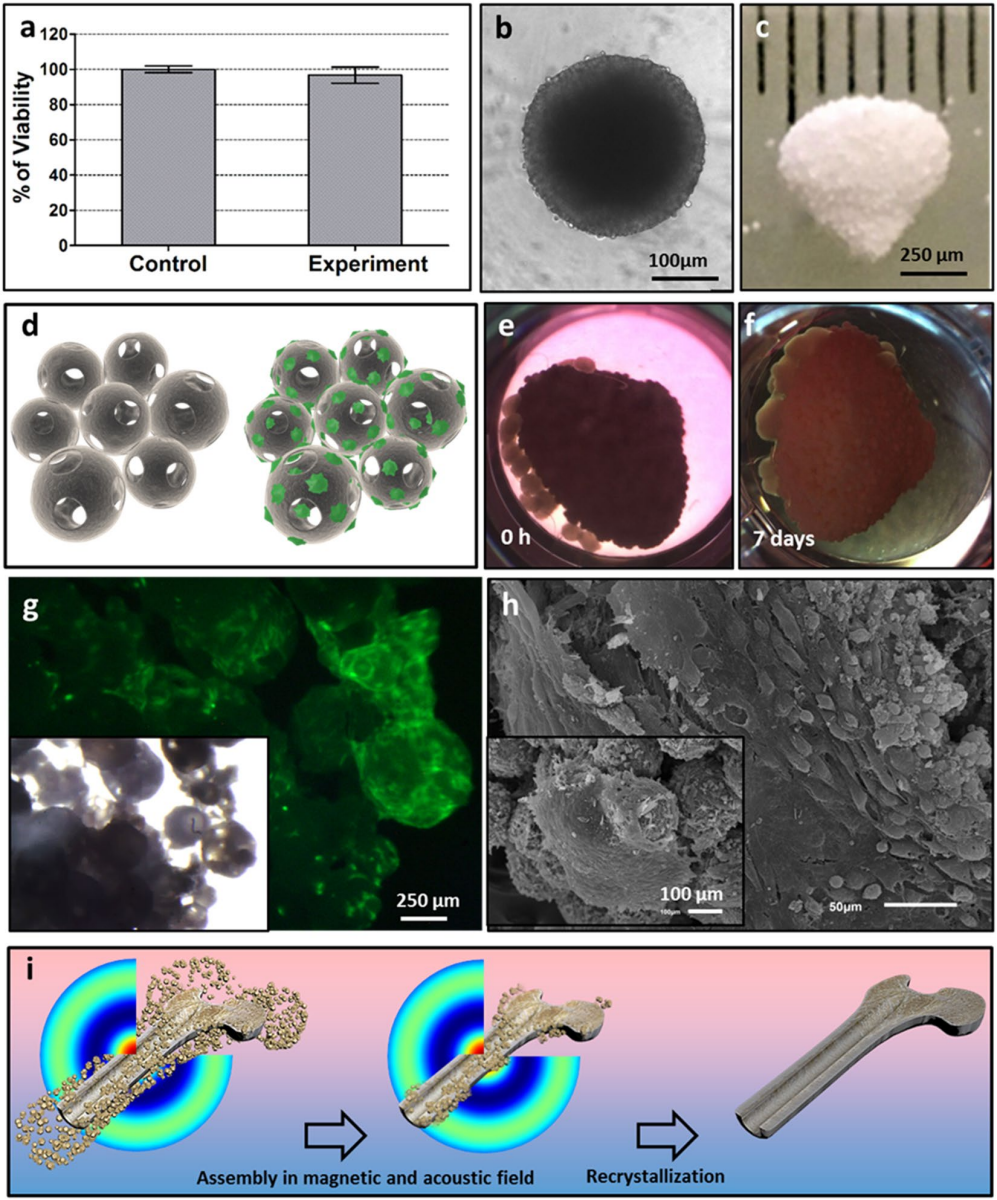
Biocompatibility of 3D scaffold and illustration of the fabrication 3D scaffold using a magneto-acoustic field. (**a**) The effect of medium after 4-days incubation with CP-based 3D scaffold on the cell viability (Alamar Blue assay, 72 h). (**b**) SHED tissue spheroid from 27000 cells in Spheroid microplate (bar 100 μm). (**c**) CP-based 3D scaffold immediately after fabrication (bar 2000 μm). (**d**) Schematic showing of SHED cells (green) attachment and migration on the surface and inside the 3D scaffold. (**e**) CP-based 3D scaffold incubated with SHED spheroids (0 h). (**f**) CP-based 3D scaffold incubated with SHED spheroids within 7 days. (**g**) Fluorescence and light microscopy images of 3D scaffold incubated with SHED spheroids (green) within 7 days. (**h**) SEM image of 3D scaffold with SHED spheroids on the surface after 7 days incubation (bar 50, 100 μm). (**i**) The schematic representation of the fabrication of a three-dimensional scaffold using a magnetoacoustic field, including the assembly and recrystallization of the scaffold.

For the assessment of 3D scaffold cytotoxicity, we used the extract-based cytotoxicity assay which evaluates the cytotoxicity of any leachable byproducts from the material. Figure 4a shows that SHED cells in culture medium pre-incubated with 3D scaffold retained 97% of viability after 72 h of incubation in the presence of extracts.

Surface properties of the 3D scaffold in regard to cell colonization were estimated by fluorescence microscopy and SEM. We used 1-day-old tissue spheroids from SHED cells (Fig. 4b) and incubated them in close contact with the 3D scaffold (Fig. 4c) for 7 days. The schematic showing of SHED cells migration from tissue spheroids and their attachment to the surface of 3D scaffold is shown in Fig. 4d. It can be seen that tissue spheroids attached to the 3D scaffold and homogeneously spread on its surface after 7 days of cultivation (Fig. 4e–h). It indicates nontoxicity and high biocompatibility of the 3D scaffold based on OCP.

## Discussion

The main goal of the present study was to prove the possibility of using the formative approach based on magnetic levitation assembly of the α-TCP particles with subsequent recrystallization into OCP considered as most favorable type of CP for bone remodeling to form 3D scaffold for bone tissue engineering^24,25^. The technology of magnetic levitation has been demonstrated previously in the number of innovative studies^18,22^. The authors have used various objects for magnetic levitation such as polystyrene beads, cells, hydrogel blocks, tissue spheroids etc. In this study we presented for the first time the magnetic levitation assembly of CP particles with the use of gadolinium salts in special buffer solution followed by recrystallization under levitation condition.

We took in account the current concepts for scaffolding include specific requirements in terms of porosity, mechanical strength, cell adhesion, biocompatibility, cell proliferation and etc. In our work, large pores effect from the particles packing. The same particle size can be free united into a body possessing the open pore fraction about 0.4 of the total body volume. The packing density matches to the coordination number (N) approximately equal to 7 in the particles unite N = 11.6(1 − Θ), where Θ is the pore fraction. N value is closely related to the coordination number in a body-centered cubic (bcc) lattice model. Based on this model, smallest pore size could be measured from simple geometric consideration of the packing density in the most dense-packed (110) plane of the (bcc) structure. This smallest pore size is about of 0.2 × 0.4 of the spherical particle diameter^26^. Therefore, the uniting of the same particles size of about 100–200 μm in diameter gives the open interconnecting pores of up to 50 ×  100 μm^2^ cross-section. This dimension correspondences to the minimum requirement in terms of porosity for bone grafting.

The compressive strength is one key parameter for bone tissue engineering constructs. 3D scaffolds had slightly low compression strength. It is about 5 MPa. However, this value of the compression strength of 3D scaffolds was still sufficient for bone tissue engineering. The preparation of OCP still presents many challenges, which among others are associated with time duration of the synthesis. Thus, some authors reported about “two weeks” and more prolonged periods^8^. However, our approach allows creation of 3D scaffolds based on OCP phases within 40 h. Moreover, according to the SEM and X-ray analysis, gadolinium salts do not remain in the structure or on the surface of the CP particles. Our results indicate that the magnetic field strongly promotes the process of growth and recrystallization of the crystals. Obtained data correlates with another research devoted to study of the influence of non-homogeneous magnetic field on protein crystal quality^27^. The authors cited work have shown that the magnetic field increases the speed of growth and improves the purity of produced crystals which can be attributed to magnetic orientation and magnetic suppression of buoyancy convection. However, the mechanisms that underlie the process of recrystallization of CP in the magnetic field are still a matter of discussion.

In addition to the speed of recrystallization, another important property of the scaffold is its porosity. The assembly of the scaffold implies the densest packing of spherical particles. The space between granules is referred to as interpenetrating pores and there is a linear correlation between interpenetrative porosity at the densest hexagonal close-packing of spherical particles and their diameters. According to our data, the diameter of interpenetrating pore comprises approximately 0.225 of the particle diameter. At the present time the application of porous ceramics with interpenetrating pores is being actively investigated as a material for filling of bone defects^28^. Interpenetrating pores are expected to have a diameter 10–135 μm to provide blood and nutrients supply to contact surface as well as facilitate the germination and anchoring of newly formed bone tissue^29–31^. The presence of pores of smaller diameters is also essential as they promote the increase in protein adsorption and adhesion of osteogenic cells. Thus, application of porous particles additionally provides the osteoconductive properties and the ceramic with bimodal pore size distribution seems to be the most preferred. In our study the fabricated scaffolds had sufficient level of porosity which could enable migration of seeded stem cells inside the porous channels. Alternatively, cells or tissue spheroid could be placed into inter- scaffold granular space.

The choice of cell type and source is an important aspect of bone tissue engineering. Nowadays, different osteoblastic cell lines and MSCs from various sources including bone marrow, adipose tissue and dental pulp are the most commonly cells used for studies on scaffolds. MSCs are cells that are naturally involved in physiological regeneration processes. It is worth mentioning that MSCs are capable of further differentiation toward osteogenic lineage which makes them, complete with OCP-based 3D constructs, promising candidates for use in tissue-engineering approaches to bone regeneration.

In the present study, we utilized primary adhesive cultures of MSCs from the pulp of deciduous teeth collected after normal exfoliation – SHED (stem cells from human exfoliated deciduous teeth). These cells are naturally involved in physiological regeneration processes and can undergo osteogenic differentiation *in vitro*. This makes them promising candidates for use in tissue-engineering approaches to bone regeneration. Thus, the fabrication of tissue engineered scaffold based on OCP and MSCs intended for regeneration of bone defect and replacement of bone tissue can be realized using our approach. To prove this statement, additional investigations of their mechanical and biological properties (including orthotopic animal *in vivo* tests) should be performed. Another fundamental question which has arisen from this study is modelling of recrystallization process of CP under the levitation condition in the non-homogeneous magnetic field which also has to be clarified in future experiments.

However, it should be noted that for fabrication of large-sized scaffolds or bone sections, it is necessary to use powerful magnetic fields, for example, generated by Bitter magnets. In such installations, in combination with the acoustic system and the visualization system of the internal structure, it is possible not only to fabricate large scaffolds, but also under the influence of the radiation force generated by the acoustic field to fabricate scaffolds with a complex internal structure (characteristic of the diaphysis or apiphysis of the bone) (Fig. 4i). The acoustic system can consist of multi-element acoustic emitters oriented in three dimensions. To ensure fabrication of complex scaffolds, for instance, system of haversian canals for subsequent vascularization, it is necessary to control the internal structure and, if necessary, make changes to the structure of the acoustic field by changing the frequency and amplitude, for example, using real-time computed tomography.

In summary, magnetic levitation technology is a promising approach and allows rapid fabrication of 3D scaffolds involving the recrystallization of OCP. Further advancement in technology of magnetic levitation assembly involving the application of acoustic and electric fields provides the possibility for rapid fabrication of engineered scaffolds with complex shape and specific macro- and microstructure. In theory, this technology offers an opportunity for rapid assembly and fabrication of tissue-engineered scaffolds based on CP and living cells. Design and development of such assemblers and biofabrication tools may contribute significantly to the field of bone tissue engineering.

## Methods

### Chemicals and reagents

Dulbecco’s modified Eagle’s medium (DMEM, Cat. No: 12491–015), fetal bovine serum (FBS, Cat. No: 16000–044), antibiotic-antimycotic (Cat. No: 15240-062), trypsin/EDTA (Cat. No: 25200-114) were obtained from Gibco (USA). EDTA (Cat. No: R080), L-glutamine (Cat. No: F032) was obtained from PANECO (Russia). High purity-grade calcium nitrate (Cat. No: 13477-34-4), ammonium carbonates (Cat. No: 506-87-6), diammonium phosphate (Cat. No: 7722-76-1), sodium acetate (Cat. No:127-09-3), glutaraldehyde (Cat. No: G5882) and resazurin sodium salt (Cat. No: R7017-5G) were obtained from Sigma-Aldrich (USA).

The production of α-TCP powder was performed according to Komlev *et al*.^19^ α-TCP powder was synthesized in an aqueous medium by slow addition of diammonium phosphate ((NH_4_)_2_HPO_4_) solution into calcium nitrate (Ca(NO_3_)_2_·4H_2_O) solution, containing ammonia solution (NH_4_OH), under constant stirring. The *pH* value of the mixture was about 7 with Ca/P molar ration of 1.5/1. After total addition of the reactants, the suspension was filtered, dried at 80 °C and sintered at 900 °C for 2 h.

To produce α-TCP particles, we used a method based on the spheroidizing of liquid drops under the action of surface tension forces using a mixed suspension of α-TCP, a binder (gelatin), and oil (Fig. S1, Supporting Information). This method allows producing porous granules of spherical shape with open pores obtained by burnout of the binder. The spherical particles produced under the action of surface tension forces were filtered on a Büchner funnel, washed free of oil, dried, and then heated to 1300 °C. α-TCP particles with mean size 250–500 μm were used for scaffolds assembly (Fig. S1, Supporting Information). The composition of α-TCP particles was confirmed by X-ray diffraction analysis (Fig. S1, Supporting Information).

Two solutions were prepared to achieve magnetic levitation assembly and recrystallization of α-TCP particles. One buffer solution (further referred to as buffer №1) was prepared by dissolving of 1.5 ± 0.1 M sodium acetate and 1.0 ± 0.01 M phosphoric acid in water. The second buffer solution (further referred to as buffer № 2) was prepared by dissolving CH_3_COONa (95.2 g) in 700 mL of distilled water (which equals 1.66 mol L^−1^) with *pH* 8.2 ± 0.2. Both buffer №1 and buffer № 2 contained 3 M Gd^3+^. In order to obtain 3 M concentration, 3 mL of 1 M Gadovist (BAYER PHARMA AG, Germany) were lyophilized by using Heto PowerDry LL3000 (Thermo Fisher Scientific, USA) and dissolved in 1 mL of buffer №1 or buffer №2.

### The magnetic setup

The fabrication process was carried out in two stages – assembly of α-TCP particles under the condition of magnetic levitation and their further recrystallization. The 3D scaffolds were assembled *via* levitational formative method by using non-homogeneous magnetic field in the presence of Gd^3+^ salts at room temperature (RT). The magnetic setup (Fig. 1a) consists of magnet holding system and 2 ring-shape NdFeB (N52) magnets. The external diameter of magnets is 85 mm; the internal diameter is 18 mm; thickness (height) is 24 mm. The distribution of the magnetic induction values in the vertical and horizontal section of working area is shown in the 3D model graph (Fig. 1b,c). Dependence of the distance from levitating assembly to center of the magnetic field on relation between magnetophoretic and gravity force acting on particles is shown in Fig. 1d.

To start the process of fabrication, the transparent syringe with capacity 2 mL (SMF, Germany) was filled with buffer № 1 containing α-TCP particles (the ratio of the mass of the particles to the mass of liquid was 1:400). After vigorous shaking the particles were distributed throughout the entire volume of liquid in the syringe. After that syringe was inserted into the hole of the magnets system, α-TCP particles started to assemble in the center of the working zone. As the result, the particles assembled into the 3D levitating scaffold under the action of magnetic forces. After α-TCP particles have assembled into the scaffold, the process of recrystallization begins. It can be separated into two steps. In the first step, the assembled α-TCP particles remained levitating in the buffer №1 for 20 h. In the second step, the syringe with 3D scaffold in buffer №1 was taken out from the printer, buffer №1 in the syringe was replaced by buffer №2, after which the syringe was placed back into the magnetic setup. After that, the scaffold underwent further recrystallization under the levitation condition for additional 20 h.

In our experiment, fluid and particle relative permeabilities are very close to 1, so magnetophoretic force acting on particles is approximately linear with difference between them. As far as *µ* > 1 for paramagnets, and *µ* < 1 for diamagnets, the difference *µ*_*p*_ − *µ*_*f*_ determines the direction of the magnetic force action. As a result, objects will be pushed out into the area with lower field strength (magnetic trap) under the action of a magnetophoretic force. In the Earth’s gravity condition the equilibration of objects occurs at a certain distance from the local minimum of the magnetic field.

### Computer simulation of magnetic field and particle assembling

The simulation of three-dimensional non-homogeneous static magnetic field in a paramagnetic medium from two permanent magnets we performed using multiphysics computational program COMSOL by finite element method. Characteristics used in simulation magnetic field were as follows: relative permeability paramagnetic medium was *µ*_*f*_ = 1.00027; and material grade of NdFeB magnet N38 (VG = 1.21 TL). The magnetic field was calculated, and then placed into this field particle trajectories equation. The transient calculation of particle trajectories was conducted using COMSOl “Particle Tracing Module”. During this calculation, the following forces were considered: magnetophoretic force based on difference between medium and particle magnetic permeabilities, drag force affecting the time of assembly, elastic force of particle-particle interaction, and gravity force. Due to low velocities of particles, the Stokes’ drag law was used to describe viscous resistance. Physical characteristics of particles the same with the CP particles were chosen: the diameter was 0.5 mm, the density was assumed as 2000 kg m^−3^, the shape of the particles was assumed to be spherical, and total number of simulated particles was 400. Despite the solid CP density is 2800 kg m^−3^, particles contained air bubbles which decrease the effective density. The features of paramagnetic liquid were found experimentally and proved to be as follows: density was 1550 kg m^−3^, dynamic viscosity was 0.01 Pa·s. The calculated velocities of particles corresponded well to the experimental data.

### Material characterization

Phase composition was analyzed by conventional X-ray diffraction (XRD) technique [Shimadzu XRD-6000 (Japan), Ni-filtered CuKα_1_ target, *λ* = 1.54183 Å]. The samples were scanned from 2θ = 3°–60° with a 0.02° step a preset time of 5 seconds.

Scanning electron microscopy (SEM) apparatus (Tescan Vega II, Czech Republic), working in secondary and backscattered electron modes, was used for materials studies.

3D scaffolds based on CP with SHED cells was fixed with 2.5% v/v glutaraldehyde/PBS, dehydrated through ethanol series and then were dried in a critical point dryer (HCP-2, Hitachi Koki Co. Ltd., Japan) and proceeded for the observation using the microscope JSM -6510 LV (JEOL, Japan).

All samples were sputter-coated using ion coater (IB-3, EIKO, Japan) with a 25 nm-thick gold layer prior imaging to impart electrical conductivity to the surfaces.

Open pore size distribution was measured using an Autoscan Qunatachrome Porometer, USA; their content was estimated by a common hydrostatic weighing in distilled water.

The compressive strength of the samples was evaluated in accordance with the ISO standard 83.100: Cellular materials. Five samples for each point were used. Compression testing was carried out using an Instron 5581 (Bucks, UK) testing machine operating at a crosshead speed of 1 mm × min^−1^.

### Cell culture

In the present study, we utilized primary adhesive cultures of MSCs from the pulp of deciduous teeth collected after normal exfoliation – SHED (stem cells from human exfoliated deciduous teeth) (Fig. S4, Supporting Information). Human deciduous teeth were collected from 3 healthy children (5–8 years old) after their normal exfoliation. Samples were stored in HBSS containing antibiotic/antimycotic (Gibco, USA) until delivery to laboratory within 24 h. Pulp tissue was mechanically extracted from the crown, disintegrated and digested in 0.1% collagenase type I solution in HBSS (Gibco, USA) for 60 min at 37 °C. SHED (stem cells from human exfoliated deciduous teeth) cells were harvested by centrifugation and suspended in the growth medium (DMEM/F12 supplemented with 10% fetal bovine serum and 100 units/ml penicillin/streptomycin (all from Gibco). The cells were expanded in 75 cm^2^ flasks under standard conditions (37 °C, humidified air containing 5% CO_2_). For passaging, the cells were detached by incubating with 0.25% v/v trypsin-EDTA solution (Paneco, Russia) for 5 min at 37 °C, suspended in growth medium and subcultured at a 1:3 ratio. Cells were free of mycoplasma contamination as verified using Hoechst 33258 (Sigma, Cat. No: 861405) staining protocol.

Primary SHED culture was analyzed by flow cytometric immunophenotyping for the presence and absence of certain markers (Supporting Information), as recommended by the International Society for Cellular Therapy (ISCT)^32^. The cells were found to be strongly positive (greater than 95%) for CD29, CD44, CD49b, CD73 and CD90 and negative (less than 2% positive) for CD34, CD45 and HLA-DR.

We also tested our cell cultures for the multipotent capacity to differentiate toward osteogenic, adipogenic and chondrogenic lineages since the ability to differentiate into three different lineages is another mandatory ISCT’s criterium for MSCs (Fig. S5, Supporting Information). Our results indicated that multipotent mesenchymal stromal cells were the predominant cells in our SHED cultures.

### Flow cytometry

Primary SHED cultures were subjected to flow cytometry analysis to determine the surface expression of CD markers whose combination typically corresponds to MSCs.

After trypsinization, 50 μL aliquots of a single-cell suspension (2 × 10^6^ cells/ml in PBS) were stained with fluorescently labeled monoclonal antibodies against CD29, CD34, CD44, CD45, CD49b, CD73, CD90 and HLA-DR (BD), according to the manufacturer’s protocol, and then analyzed using a FACS Aria III flow cytometer (BD).

### Multilineage differentiation

#### Osteogenic differentiation

Cells were cultured in 6-well plates. When their confluence reached 70–90%, the growth medium was replaced with the osteogenic differentiation medium (DMEM containing 2 mM L-glutamine, 100 U/ml penicillin, 100 U/ml streptomycin (all from Gibco), 0.2 mM ascorbic acid, 10 mM glycerophosphate and 0.1 μM dexamethasone (all from Sigma)). The medium was refreshed every three days. After 14 days of differentiation, cells were fixed with 4% paraformaldehyde and stained with 2% alizarin red (Sigma) to detect mineral (calcium) deposition. The level of alkaline phosphatase activity was evaluated using the StemGent Alkaline Phosphatase Staining kit (ReproCELL), following the manufacturer’s protocol.

#### Adipogenic differentiation

Cells were cultured in the adipogenic medium (DMEM supplemented with 10% horse serum, 0,5 mM isobutylmethylxanthine and 60 μM indomethacin (all from Sigma)). The medium was refreshed every 3 days. After 14 days of differentiation, cells were fixed with 4% PFA and stained with Oil Red O (Sigma) to detect intracellular lipid droplets.

#### Chondrogenic differentiation

Chondrogenic differentiation was performed in tissue spheroids. Cells were detached and brought into suspension by incubating in the mixture (1:1) of 0.25% trypsin and Versene (Paneco, Russia) for 5 min at 37 °C, harvested by centrifugation, and resuspended to a concentration of 10^6^ cells/ml in the above growth medium. Aliquots (100 µl) of cell suspension were placed in the wells of a 96-well ultra-low attachment (ULA) culture plate (Corning) and incubated for 24 h under standard conditions to achieve spheroid formation. Afterwards the medium was changed with chondrogenic inducing medium (DMEM supplemented with 1% FBS, 10% ITS + Premix Supplement, 1% sodium pyruvate (all from Gibco), 0.1 μM dexamethasone (Sigma), 50 μg/ml L-ascorbic acid (Sigma) and 10 ng/ml transforming growth factor-β1 (Peprotech)). The medium was replaced every 3 days. After 21 days of differentiation, the resulting spheroids were fixed with 4% paraformaldehyde and subjected to alcian blue staining for glycosaminoglycan (GAG) content in their extracellular matrix.

### Formation of tissue spheroids using corning spheroid microplates

The tissue spheroids were formed using Corning spheroid microplates (Corning, Cat. No: 4520) according to the manufacturer protocol. Briefly, cells in monolayer with 95% confluence were rinsed by EDTA solution, harvested from the substrate by 0.25% trypsin/0.53 mM EDTA and then resuspended in cell culture medium. The cells concentrations were 2.7 × 10^5^ per milliliter. 100 μl of cell suspension were delivered into the wells of Corning spheroid microplates. Corning spheroid microplates were incubated at 37 °C in a humidified atmosphere with 5% CO_2_ for 24 h.

### Cytotoxicity assay of 3D scaffold based on calcium phosphate

The extract testing was used for the cytotoxicity assessment. 3D scaffold based on CP was culture medium soaked at 37 °C for 4 days. SHED cells were plated in 96-well culture plate at a concentration of 1 × 10^4^ cells/well. Each well contained 100 µl of cell suspension and the plate was incubated for 24 h at 37 °C in a humidified atmosphere with 5% CO_2_ to obtain a pre-monolayer culture. Then, 200 µl of culture medium pre-incubated with 3D scaffold was inserted to experimental wells and 200 µl of fresh culture medium was inserted to control wells. The plate was incubated for additional 72 h at 37 °C in a humidified atmosphere with 5% CO_2_ following which 200 µl of supernatant was aspirated and 10 µl of 0.02% resazurin solution in culture medium was added to each well of plate. The plate was returned to CO_2_-incubator for 6 h and then fluorescence was recorded at excitation wavelength of 555 nm with emission detected at 580 nm using VICTOR X3 Multilabel Plate Reader (Perkin Elmer, USA). The wells contained cell culture medium without any cells were used to assess background signal.

### Biocompatibility of the 3D scaffolds

The biocompatibility of the 3D scaffolds was investigated in relation to SHED tissue spheroids by fluorescent microscopy (NIKON SMZ18, USA) and SEM (JSM-6510 LV, JEOL, Japan). 1-day-old tissue spheroids from SHED cells were incubated in close contact with CP-based 3D scaffold for 7 days. Cell viability was monitored using the Live/Dead Cell Double Staining Kit (Sigma-Aldrich, USA) according to the manufacturer’s protocol. This assay was used to visually determine if cells within tissue spheroids remained viable after cultivation with CP-based 3D scaffold. Briefly, after 7-days cultivation, 3D scaffold with 1-day-old tissue spheroids from SHED cells was incubated with a solution containing Calcein AM and propidium iodide (PI) at 37 °C for 30 min. After washing with PBS, tissue spheroids were imaged by fluorescent microscopy (NIKON SMZ18, USA).

### Data analysis

Statistical data was analyzed using GraphPad Prism software (GraphPad Software, Inc., La Jolla, CA) and represented as mean ± S.E.M. The Analysis of Variance (ANOVA) test was used to find the significant differences between the means of the three groups with *P* < 0.0001.

## Supplementary information

Supplementary Information

Supplementary Information
