# Supplementary material for: Fabrication of calcium phosphate 3D scaffolds for bone repair using magnetic levitational assembly
Source: Sci Rep. 2020 Mar 4;10:4013. doi: 10.1038/s41598-020-61066-3 (PMC7055252; doi:10.1038/s41598-020-61066-3)
Supplement: Supplementary file 1 — Supplementary Information [file 41598_2020_61066_MOESM1_ESM.docx]

**SUPPORTING INFORMATION**

**Fabrication of calcium phosphate 3D scaffolds for bone repair using magnetic levitational assembly**

**Vladislav A. Parfenov^1,2*^†, Vladimir A. Mironov^1^†, Elizaveta V. Koudan^1^, Elizaveta K. Nezhurina^3^, Pavel A. Karalkin^1,3^, Frederico DAS Pereira^1^, Stanislav V. Petrov^1^, Alisa A. Krokhmal^1^, Timur Aydemir^1^, Igor V. Vakhrushev^1,4^, Yury V. Zobkov^2^, Igor V. Smirnov^2^, Alexander Yu. Fedotov^2^, Utkan Demirci^5^, Yusef D. Khesuani^1^, Vladimir S. Komlev^2^**

^1^ Laboratory for Biotechnological Research “3D Bioprinting Solutions”, Moscow, Russia.

^2^ A.A. Baikov Institute of Metallurgy and Material Science, Russian Academy of Sciences, Moscow, Russia.

^3^ P.A. Hertsen Moscow Oncology Research Center - branch of National Medical Research Radiological Center, Moscow, Russia.

^4^ V.N. Orekhovich Institute of Biomedical Chemistry, Moscow, Russia.

^5^ Stanford University, Department of Radiology, Stanford, CA, USA.


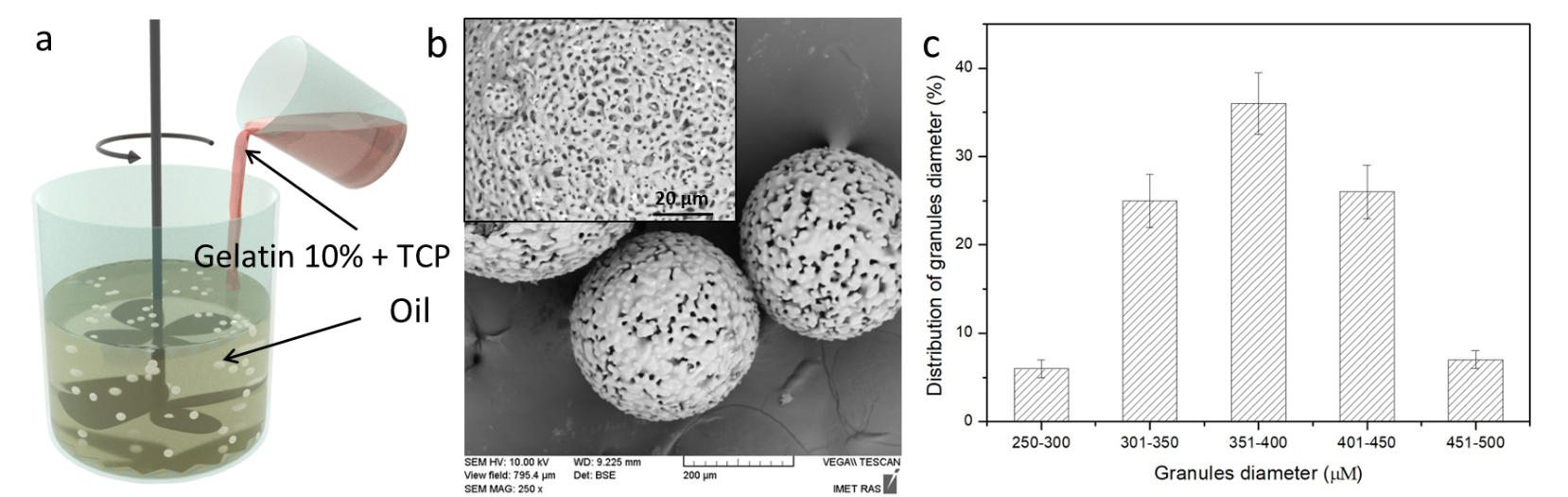


**Figure S1.** Fabrication of α-TCP particles. (a) Schematic showing the fabrication process of α-TCP particles. (b) SEM image of α-TCP particles (bar 200 μm and 20 μm). (c) Distribution of diameter of α-TCP particles.


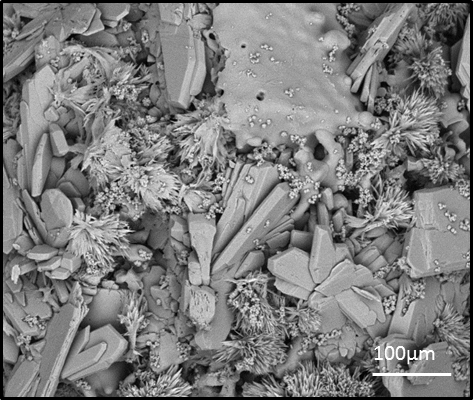


**Figure S2.** SEM image of scaffold fabricated under the same conditions without application of the magnetic field (bar 100 μm).


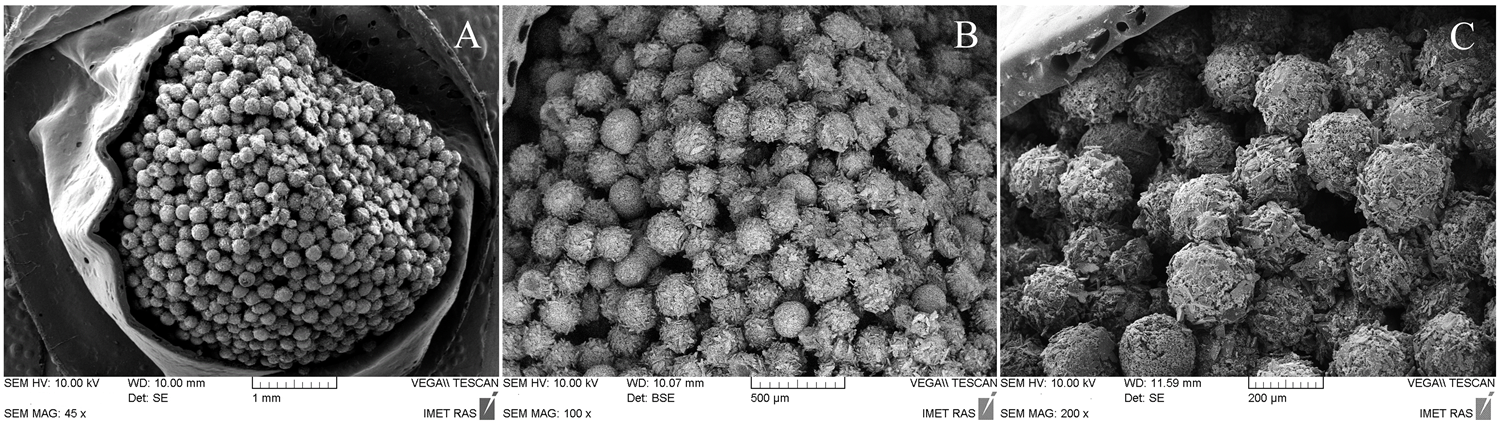


**Figure S3.** General view of the printed construct. (a) ×45. (b) ×100. (c) ×200.


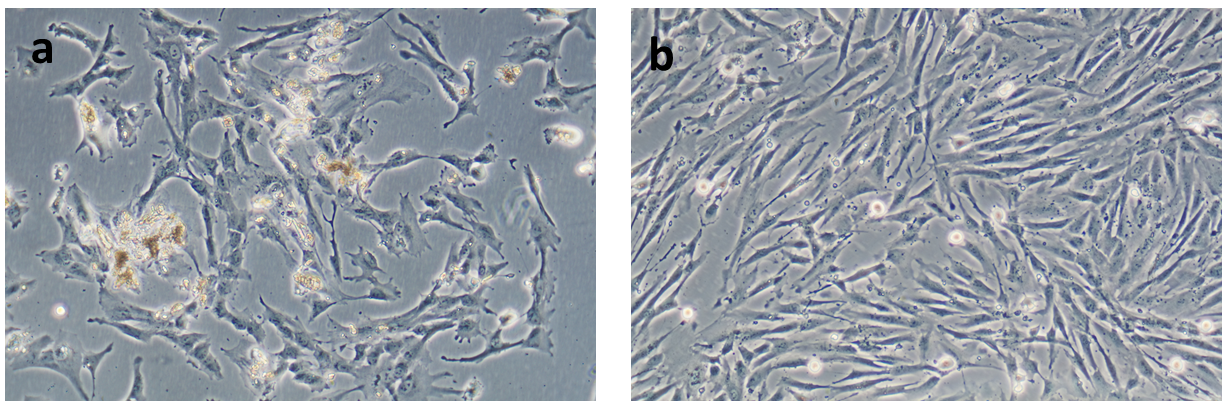


**Figure S4.** Obtained primary SHED cultures. (a) – prior to first passaging. (b) – at the passage 4. Phase-contrast microscopy; magnification: ×100


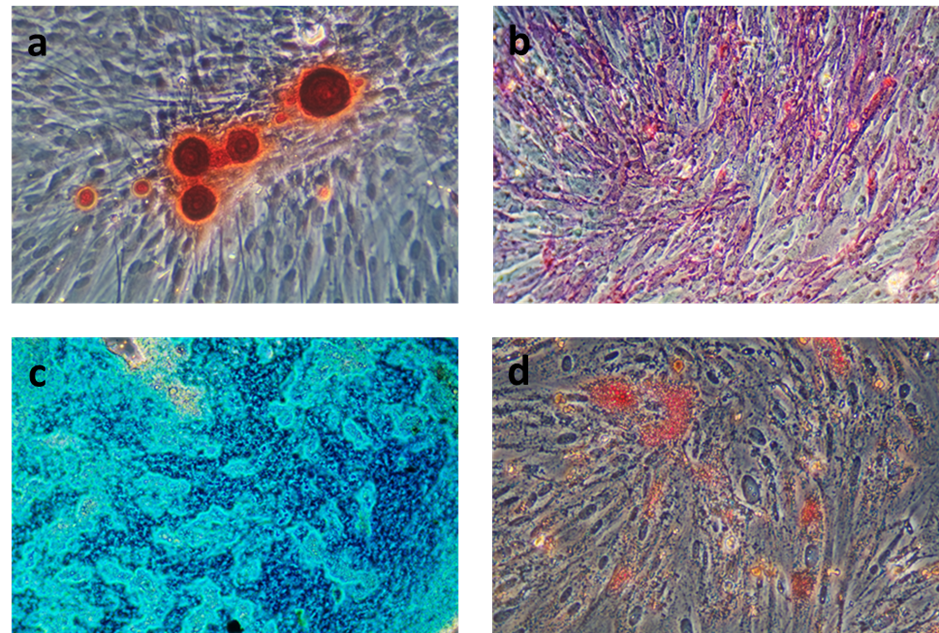


**Figure S5.** SHED multilineage differentiation. MSCs in our SHED cultures were functionally verified, after culturing for two weeks in differentiation media. (a) Osteogenic differentiation was detected by Alizarin Red staining to visualize calcium deposits and also with (b) alkaline phosphatase assay. (c) Chondrogenic differentiation was detected by Alcian Blue staining to visualize the production of glycosaminoglycan-rich matrix. (d) Adipogenic differentiation was detected by Oil Red O staining to visualize intracellular lipid vacuoles. Shown are representative light photomicrographs; magnification: ×200 (a, d) and ×100 (b, c).


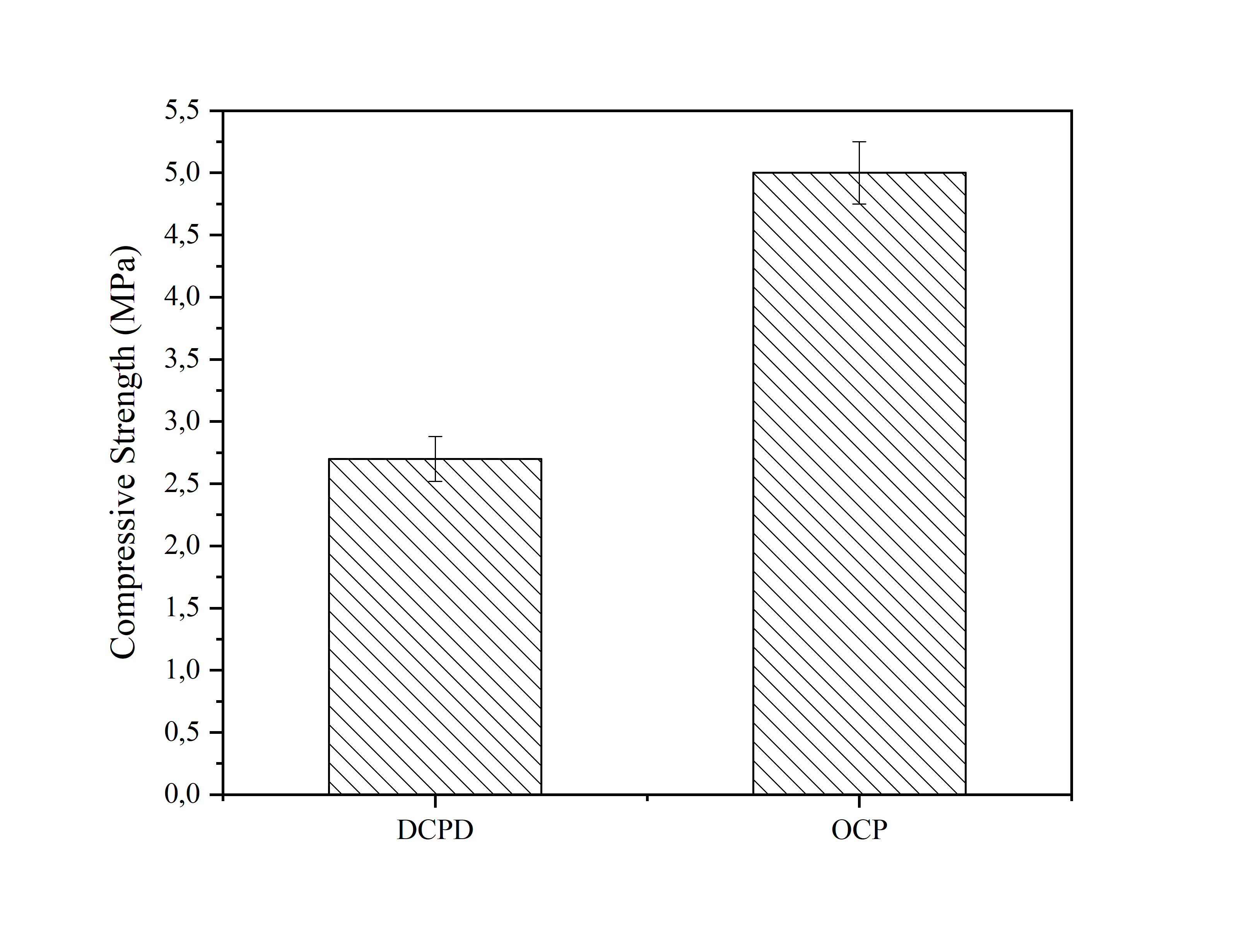


**Figure S5.** Compressive strength (a) of 3D scaffold after phase 1 of magnetic levitation fabrication (20 h in buffer №1, RT), and (b) of 3D scaffold after phase 1 (20 h in buffer №1, RT) and phase 2 (20 h in buffer №2, RT) of magnetic levitation fabrication.

**Video S1** – The assembly of α-TCP particles in the paramagnetic medium (concentration of gadolinium salt 3M) under the condition of magnetic levitation in non-homogeneous magnetic field.
